# Supplementary material for: Long-term soil warming decreases microbial phosphorus utilization by increasing abiotic phosphorus sorption and phosphorus losses
Source: Nat Commun. 2023 Feb 16;14:864. doi: 10.1038/s41467-023-36527-8 (PMC9932148; doi:10.1038/s41467-023-36527-8)
Supplement: Supplementary file 1 — Supplementary Information [file 41467_2023_36527_MOESM1_ESM.pdf]

Supplementary Fig. 1 | X-ray diffractograms

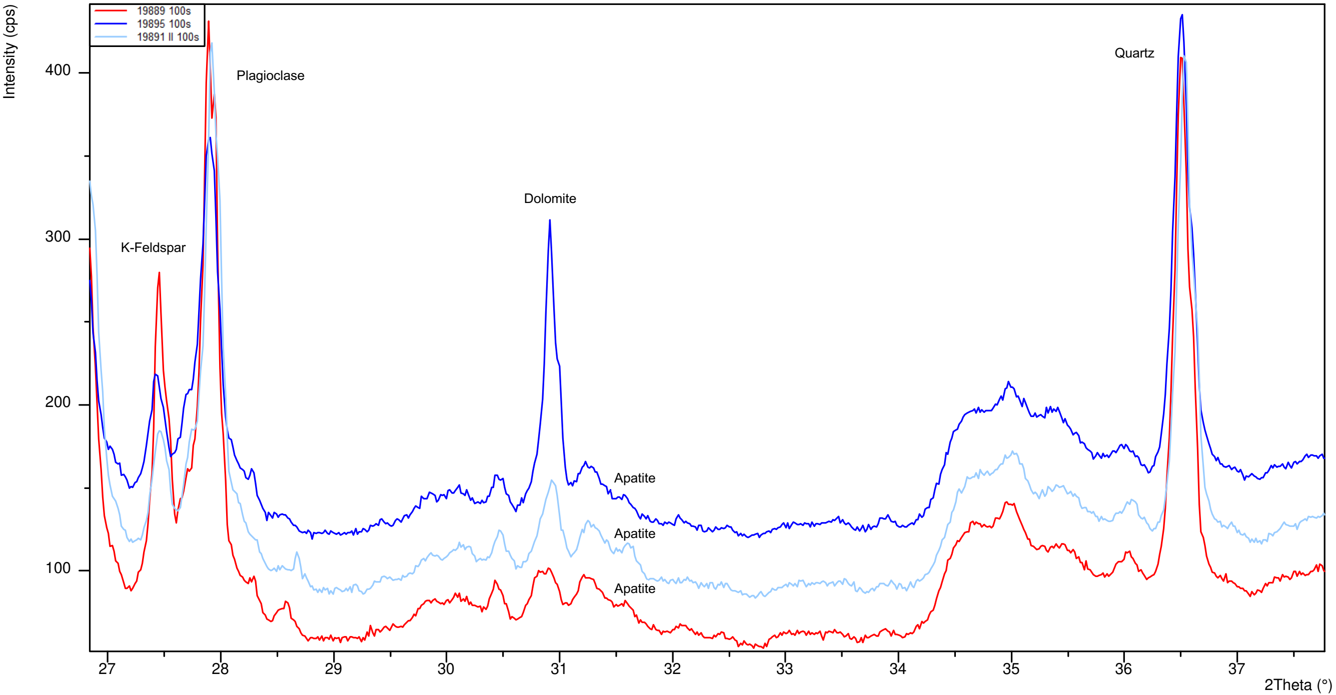

X-ray diffractograms from three out of twenty-four samples are presented for visibility reasons. Other diffractograms are available upon request.
